# 

**Published:** 1893-01-28

**Authors:** 


					282 THE HOSPITAL. Jan. 28, 1893,
Notices of Births, Deaths, and Marriages, are inserted in
this column at a charge of 2s. 6d. (or an announcement not exceeding
four lines.
NOTICE TO CORRESPONDENTS.
All MS., Letters, Books for Review, and other matters intended fot the
Editor should be addressed Thb Editob, The Lodge, Pobchbsteb
Square,London, W.
The Editor oannot undertake to retnrn rejected U.S., even when
accompanied by stamped directed envelope.
Notice to Advertisers and Subscribers.
All Advertisements, Orders for OopieB of Thb Hospital, and Business
Oommnications should be addressed to The Publisher, not to the Editor,
Thb Hospital, 140, Strand, W.O.
The Oost of Subscription, post free, is as followsPer week, 2}d. j
per quarter, 2s. 6d. ; per half-year, 5s.; per annum, Ss. 6d.
Foreign s?Per annum, 10s. 8d.; payable, in all cases, in advance.
"Bnrdett's Hospital Annual," 1893.
Fourth year of publication. 700 pp. Boards, gilt lettering. A most
complete guide to British, Oolonial, Indian, and American Hospitals,
Dispensaries, Nursing and Convalescent Institutions, and Asylums.
Price5s. Published by Thb Scientific Press (Limited), 140, Strand,
W.O.
NOTICE.?The Index for Vol. XII. fending- September
1882) is on sale at the Office of this Journal, price
2fcd., post free.
Scraps and Gleanings.
A snowball scheme is on foot in connection with the fands to to
raised for the Cardiff Infirmary
Baron Hmscu has sent ?300 in aid of the National Society for the
Prevention of Cruelty to Children.
In addition to the donations already announced, Baron Hirech has
sent ?1 200 to St. Mary's Hospital, Paddington.
The Duke'of York has announced hisUntention of becoming an annual
sub criber to the Boyal Naval Exhibition Fund for the widows and
orphans of seamen and marines.
Mr. G. R. Ccndell, M.K.O.S., L.S.A., of Brunswick House, Kew,
has been aDoointed one of the medical officers 10 sea out-patients of the
Richmond Hospital, vioe Mr. J, A. Nowell.
It is considered necessary to erect a temporary hospital in Manchester
to meet the spread of the small-pax epidemic, 70 cases of that disease
being reported from the Monsall Hospital,
Monsieur Hatikine, of the Pasteur Institute, is shortly going to
India in the hopes of applying praotically his method in the saving of
life of vicsims to endemic cholera in British India.
The Prejton Sanitary Committee recommend the erection of a hos-
pital for c 'eei of cholera in vessels frequenting the port. The estimate,
inclusive of drainage, is estimated at about ?900,
The General Committee de la Seine havo decided to give an annual
subEO-iption to the Pasteur Institute, as an accord of honour to the
manifestation on the 27th iu the great dootor's honour.
At the last meeting1 of the subscribers to the Coldstream Cottage
Hospital, it was reported that the ?1,000 required for the Tweed Bed is
now made up, Lidy Home raising the sum through har own personal
exertions.
The ninth annual report of the Totteridge Cottage Home is a hisrhly
satisfactary one. Sixty-nine children, it states, have been reoeived
during the past year, of whom 37 were on " frea letters," 29 at 53., and
three at 3a. per week.
The Queen's Jubilee Hospital at Earl's Court is having a bazaar
arranged for next May in aid of its funds. This hospital is unendowed.
S nee its commencement over 1>000 in-patients, and over 12J.C00 out-
patients hive been treated.
Miss Maet Elizabeth Gabrett, of Baltimore, has contributed a
large sum of money to the John Hopkins Univereity, thu3 completing
the required sum needed to open the Medical School, where women ara
i o be admitted on equal terms with men.
A New Year's treat was given at Bradford Infirmary when, in re-
sponse to the invitation of the House Committee, a large gathering
collected to witness the annual distribution of toys and other gifts
amongst the children who are undergoing treatment there.
Mr. Frances and Mr. James Reckett have proved themselves most
generous benefactors to Hull, having, at the cost of some thousands of
pounds, bought the Queen's Hotel at Withernsea, for the pnrposa of
establishing a convalescent home in connection with the Hull Royal
Infirmary.
A laroe public dinner in aid of the Building Fund of the Great
Northern Central Hospital, at which H.R.H. the Duke of York wiH
preside, will be held next spring. Tbe completion of the hospital, with
the necessary furniture, will cost ?27,000, towards which sum only
JE8.000 has been received.
At the annual meeting of the Riyal Infirmary. Edinburgh, the Lord
Provost pointed out that* the decrease of ?8i9 derived from the
students' tickets was owing to the imposition of a five years instead of
a four years* curriculum, which was having the cffect of checking the
number of students in all the medical schools.
Vlll
THE HOSPITAL. Jan. 28, 1893'
The Student of Medicine.
1 All oommnnioationa for this Supplement should be addreued to Thi Editor of Thb Hospital, at 140, Strand, with the worda," Stndc
Oolnmn," written in the left hand top corner of the envelope.!
VACANCIES.
The following1 vacancies are announced this week, the amount of
salary in each oase being given after the name of the institution:
Resident Medical OfSoers.
Albany General Hospital, Grahamstown, Santh Africa. Resident
Medical Officer. Salary ?250 per annnm, with unfurnished rooms.
Further particulars to ba obtained of Dr. 0. W. Cathoirt, Royal
Infirmary, Edinburgh.
Bury Dispensary Hospital, Bury, Lancashire. Junior House Surgron.
Doubly qualified. Salary ?30 per annum, with board, residence,
and attendance: Applications to the Secretary, Henry Webb,
Brentwood, Bury, by February 1st.
Chichester Infirmary, Ohiohester. House Surgeon. Salary ?80 per
annum, with board, lodging; and washing. Applications to B. E.
Street, Secretary, by March 1st.
Dundee Royal Infirmary. Medical Superintendent. Applications to
the Secretary, D, Gordon Stewart, Solicitor, Dundee, by February
8th.
Bast London Hospital for Sick Children, Glamis Road, Shadwell, E.
House Surgeon. Board and lodging provided. Applications to the
Secretary by January 25th.
General Hospital, Birmingham. Two Assistant House Surgeons.
Appointments for six months. Board, residence, and washing
provided. Applications to the House Governor by January 28 th.
Ripon Dispensary and Cottage Hospital, Ripon. Resident House
8urgron and Dispenser, Unmarried. Salary ?70 par annum, with
board and lodging. Applications to F. D. Wise, Honorary
Secretary.
Royal South Hants Infirmary, Southampton. Assistant House Surgeon.
Board and rooms provided. Applications to T. A. Fisher Hall,
Secretary, by January 30th.
Btratford-on-Avon Rural Sanitary Authority. Medical Officer of Health.
Salary ?110 per annum. Applications to John Oharlas Warden, 9,
Guild Street, Stratford-on-Avon, by January 21st.
West Herts Infirmary, Hemel Hempstead. House Surgeon and Dis-
penser, doubly qualified, unmarried. Salary ?100 per annum, with
board, furnished rooms, light, fire, attendance, and washing.
Applications to the Secretary by January 31st.
Hon-Resident Modioal Offioera.
Birmingham and Midland Hospital for Women. Vacacey ary
Honorary Acting Oat-patient Staff. Applications to the n ty
Secretary, Mr. Russell Jolly, 109, Colmore Eoad, Birming
February 1st. ?l00 p?r
Corporation of Norwich. Medical Officer of Health. t0
annum. Applications, endorsed "Medical Officer of _rT 2Stb>
Geo. B. Kennett, Town Clerk, Guild kail, Ncrwch, by "'an'L 'or?ry
Royal South London Diapen?ary, St. Georgo's Cross, S.E. J* * ^
Surgeon. Applications to the Co mmittee of Manage
January 28th. ,-g A
University College of South Waist and Monmouthshire, Oar ^ .n
Professor of Anatomy, and a Professor of Physiology. j>egistraTi
each case ?350 per annum. Applications to Ivor James, ? ?
by February 10th,
PASS LIST.
Society of Apothecaries of London, Deosmber, 189-
The following candidates paused invajs??
Sueoiet.?R. H. Calvert, Leeds Yorkshire College ; H.
St. Mary's Hospital; H. W. Clarke, St, Mary's Hospital; B. j. B.
Charing Cross Hospital; F. J. Godwin, Charing Cross SosP1^ .
Henry, St. Bartholomew's Hospital; G. M. Hethgrington, . J.
lege; G. Higginson, Cambridge University and London HosP1
Joule,London Hospital; J. Kennedy, London Hospital; E. C. ?
St. George's Hospital; G. Martyn, King's College; F. 8. P** jj9ry''
burgh; E. Le F. Payne, St. Mary's Hospital; W. F.Peaoock.S ? ^_ g.
Hospital; A. L. Roper, Cambridge Univeriity and Guy's pjtal;
Smith, Middlesex Hospital; T. E, Smnrthwaite, St. Mary'B olJfirinff
A. B. Sturges, Leeds Yorkshire College; J. M. Swanson, ^ T,
Cross Hospital; S. W. Thompson, Charing Cross Hospital; ' ffeni
White, St. Bartholomew's Hospital; F. D. Woolley, Manohee
College. rryi gt.
Medicine, Fokbnsio Medicine, and Midweek?.?R? "6 J),
George's Hospital; H. H. P. Oottan, Westminster Hospital;
28,1893. THE HOSPITAL
IX
THE STUDENT OP MBDIOIM"B?(continued).
T?^?B>Midd]oS w ? N* J?bnson, Royal Free HoBpital; A. G.
tr Tomi;- ?s ~ BPital; A. L. Knapman, Manchester Owens College ;
MPita1, George's Hospital; R. B. Williams, St. Thomas s
Vitul; ipI1?s and Foeensic Medicine.?H. F. Ealand, St. Mary's Hos-
p0^aud Eai'ubnrph and St* Mary's HosPital 5 F- s- Park> Liver'
a^0ll?ssir itrP Mi?wipebt.-R. Evans, University ColIfge.
vy's Hoar^fMcinb.-R. S. Fairbank, King's College; J. H. Boberis,
^Jlchestep n?' A-R?*>inson, Leeds Yorkshire College ; H. A, Walker,
^IDWipEnv ?olle*e 5 W. tt. Wil ey, St. Mary's Hospital.
pT? Messrp TT ' L" Godwin, Edinburgh.
;aJTie, p"'?"Otty. Calvert, Foulds, Henry, Joule, Little, Martyn,
i,??linson wt-2 JL^er, Roberts, 8. rirn it a, Swansou, Thompson,
j Society_ 1 Wiliey, and Williams was granted the diploma of
fining- Board in England, by tho Royal Colleges of
? follow Physicians and Surgeons.
J? Asajqm gentlemen r.assed the second examination of the Board
?.ay> JanUarv J!? Physiology at a meeting of the examiners on Mon-
JDd?nts of nt V. ^ Copland, W. Batement, and W. L. Christie,
?V 0. Aanlw?g0.vni,rersity. New Zealand, and London Hospital; W.
? ^Wdlegpt n London Hospital; H. G. Cribb and A. ?. Lawrencs,
E. J t>? 5ltal i J. W. Maskell, of University College, Liver-
?agoa Ooij" e^s, of Masan College, Birmingham; T. E. Price, ef
y^oloBv. t* /?lrmiDgliam, and Mr. Cooke's School of Anatomy and
a' Grant U,alj?ia. of the University of Malta ; N. B. D*rabseth,
j'fover, of n? College, Bombay, and Aberdeen College; J. M. K.
fiscal ?ens College, Manchester; J. H. Ferguson, of Ledwioh
?; Qneen'g rvV,aild the Catholic University, Dublin ; T. G. D. Cooper,
5jPital. uou?ge. Cork; and W. F. Evans, of St. Bartholomew s
te^Teni^anrtE rt g??tlemen passed in Anatomy only : H. Hallam, E. D.
RW^iatt8 and ?' m' Jlory? ?' the Medioal School. Sheffield; S. W.
0?art, of n J?nes, of University College, Liverpool } 0. H.
V?bb Oolwf ??? ^e* Birmingham ; T. Jones and E. Fielden, of
^nrridee ?; f ?n?t"?ter; A. E. Sears, of London Hospital; and H.
? fotfni? Km? 8 College.
rj' A. x t ?n? gentlemen passed in Physiology only: A. E. Little
?? St. H'ar,?ch?0re. of Yorkshire College, Leads; E. H. Milson,
^latol, hospital; R. E. Jennings, of the Medical School,
^ratt, of iviirMi 00^?*B School of Anatomy and Physiology; T. T.
&1! w fThi eeex Hospital; E. Folliott, of St. Bartholomew's Hos-
tester; A n?#'?a? and W. H. Tomlinson, of Owens College, Man-
*?Ui candiiW? ' Sutcliffe, of Mason College, Birmingham.
5 twelve in pk ^?76 'eferred in both subjects; six in Anatomy only ;
pasted in AW?^SIology on,r-
. AT0MT and Physiology on Tuesday, January 10th : A.
Marriott, T. H, 0, Stevenson, and J. H. Oook, of University Collet?,-
G. H. Hunt, of St. Mary's Hospital; T. H. Boyd, of Melbourne Un 1
versity ; F. D. S. Fayrer, of Cambridge University and Charing' Gross
Hospital; A. H. H. Huokle, Walter C. Pakes, of Guy'a Hospital ?, 0. B.
Statham, of Grant Medical College. Bombay, and Guy's Uocnital; B.
W. H. Groves and F. A. Smith, of St. Bartholomew's Hospital; w'. H."
Kenrick, of London Hospital; A. J. Eastcott and E. L. Hunt, cf" St!
Georgo's Hospital; H. 0. Crouch, of St. Thomas's Hospital ; R*
Moreton, of St. Thomas's Hospital and Mr- Cooke's Sohool of Anatomy
and Physiology; U. *V. N. Miles, of King's College ; and H. P. Noble,
of Middlesex Hospital.
Passed in Anatomy only : J. P. Prell, of London Hospital.
Passed in Physiology only : D W. fT. Latham and (J. F. Le Sago, of
London Hospital; H. J. Riohards and B. W. Holmes, of St. Bartholo-
mew's Hospital; O. Sapara, of St. Thomas's Hospital ; W. D. Watson
and B. T. Inkson, of University College; and 0. A. A. Coulthard, of
St. George's Hospital.
Twelve candidates were referred in both subjects, five in Anatomy only,
and five in Physiology only.
Passed in Anatomy aud Physiology on Wednesday, January 11th:
H. Rawsthorne, of University College ; R. A. L. Hill find P. S. Crookes,
of St. Thomas's Hospita!; A. Chopping, of St. Thomas's Hospital and
Mr. Cooke's School of Anatomy und Physiology; J. Moore and L. A.
Pau', of Guy's Hospital; L. A. Grimes, of St. George's Hospital and
Mr. Cooke's Sohool of Anatomy and Physiology; S. Wicks, of Cam-
bridge University and London Hospital; F. L. Dodd, of Middlesex
Hospital; J. M. G. Swainson, of Westminster Hospital; and J. Gund-
lach, of St. Bartholomew's Hospital.
Passed in Anatomy only : F. V. Bioe, W. A. Bramsden, H. G. Berry,
and T. Martin, of St. Bartholomew's Hospital; A. W. Robertson, of
St. Bartholomew's Hospital and Mr. Cooke's Sohool of Anatomy and
Physiology; J. G. Heath, of St. Mary's Hospital; F. R. Baker, of
London Hospital and Mr. Cooke's School of Anatomy and Physiology;
and R. E. De Martini, of King's College.
Passed in Physiology only : H. R. Emms, A, Brebner, and F. H. H.
Anning, of University College; L. 0. Dillon, of King's College; M. J.
Ryan and W. H. Farmer, of St. Bartholomew's Hospital; W. K. Hay-
Coghlan, of St. Mary's Hospital; and H. E. Dixon, of London
Hospital.
Twelve candidate! were referred in both eubjects, five in Anatomy
only, and eight in Phjsiology only.
Boyal College of Surgeons of England.
The following gentleman, having previously passed the neoessary
examinations, and having now conformed to the bye-laws and regula-
tions, was at the Quarterly Meeting of the Council on Thursday,
January 12th, admitted a Member of the College : S. W. Thompson,
L.S.A,, Charing Cross Hospital.

				

